# Molecular docking and dynamic simulation of conserved B cell epitope of SARS-CoV-2 glycoprotein Indonesian isolates: an immunoinformatic approach

**DOI:** 10.12688/f1000research.54258.1

**Published:** 2021-08-16

**Authors:** Fedik Abdul Rantam, Viol Dhea Kharisma, Christrijogo Sumartono, Jusak Nugraha, Andi Yasmin Wijaya, Helen Susilowati, Suryo Kuncorojakti, Alexander Patera Nugraha

**Affiliations:** 1 Research Center for Vaccine Technology and Development, Institute of Tropical Disease, Universitas Airlangga, Surabaya, East Java, Indonesia; 2 Virology and Immunology Laboratory, Department of Microbiology, Faculty of Veterinary Medicine, Airlangga University, Surabaya, East Java, 60132, Indonesia; 3 Biology Department, Faculty of Mathematic and Natural Sciences, Universitas Brawijaya, Malang, East Java, Indonesia; 4 Anasthesiology and Reanimation Department, Dr. Soetomo Gerneral Hospital and Faculty of Medicine, Universitas Airlangga,, Surabaya, East Java, Indonesia; 5 Clinical Pathology Department,, Dr. Soetomo Gerneral Hospital and Faculty of Medicine, Universitas Airlangga, Surabaya, East Java, Indonesia; 6 Faculty of Medicine, Universitas Airlangga, Surabaya, East Java, Indonesia; 7 Department of Veterinary Anatomy, Faculty of Veterinary Medicine, Universitas Airlangga, Surabaya, East Java, Indonesia; 8 Orthodontics Department, Universitas Airlangga, Surabaya, East Java, 60132, Indonesia

**Keywords:** COVID-19, SARS-CoV-2, Infectious Disease, Immunoinformatic, spike glycoprotein, conserved region.

## Abstract

**Background:** An immunoinformatic approach may be useful to investigate the conserved region in the spike glycoprotein of severe acute respiratory syndrome coronavirus 2 (SARS-CoV-2) Indonesia isolates. The aim of this study was to investigate Indonesian SARS-CoV-2 isolates based on B cell epitopes by targeting the conserved regions in the spike glycoprotein to trigger increased multi-variant virus neutralization and memory response for the development of vaccine seed candidates.

**Methods:** SARS-CoV-2 spike glycoprotein gene sequences originating from Indonesia were compared with Wuhan (China), the United Kingdom, South Africa, India, the United States, and Brazil isolates obtained from the NCBI and GISAID databases. The recognition of antigens was carried out directly using B cells through the B cell receptor (BCR). An indirect B cell activation by Cluster of Differentiation (CD)4+ T cells and major histocompatibility complex (MHC)-II was predicted through the binding with human leukocyte antigen (HLA) based on IC _50 _value. In addition, vaccine allergenicity and toxicity were investigated. During the molecular complex examination, the 3D peptide structure was investigated and the lowest amount of energy formed when the vaccine candidate peptide bound to BCR and MHC-II was calculated.

**Results:** As a result, the spike glycoprotein sequences of Indonesian SARS-CoV-2 isolates had conserved regions which were very similar to reference countries such as China, the United Kingdom, South Africa, India, the United States, and Brazil.

**Conclusion:** It was predicted that the conserved regions could be identified as the epitope of B and T CD4+ cells that produced the peptides for vaccine candidate with antigenic, non-allergen, and non-toxic properties.

## Introduction

Coronavirus disease 2019 (COVID-19) is caused by the severe acute respiratory syndrome coronavirus-2 (SARS-CoV-2). First identified in December 2019 in Wuhan, China, it then spread around the world and became a global health burden. COVID-19 patients may experience headaches, dizziness, coughing, and a loss of their sense of smell. ^1^ The virus is spread between humans when an infected individual expels droplets and airborne particles which come into contact with a healthy person. SARS-CoV-2 is a virus with RNA as its genetic material, an envelope, and in about 96% of COVID patients it is identified as BatCoV RaTG13. ^2^^,^^3^ This virus consists of thousands of variants divided into several clades. The most well-known variants are Alpha (B.1.1.7), Beta (B.1.351), Gamma (P.1), Delta (B.1.617.2), and Epsilon (E484K, B.1.429 & B.1.427). ^4^ The emergence of SARS-CoV-2 variants causes an increase in the rates of infection, reinfection, and avoidance of neutralization from the vaccine antibodies. ^5^

The COVID-19 pandemic has extended to all parts of the world including countries in the Southeast Asia region, especially Indonesia. The number of SARS-CoV-2 cases has increased rapidly in Indonesia, at around 1000 new cases per day since 2020. As of now, the distribution of SARS-CoV-2 vaccination in Indonesia still totally depends on foreign production. Thus, the development of domestic vaccines is necessary. ^6^ In addition, the domestic vaccine development uses various Indonesian SARS-CoV-2 isolates from the NCBI and GISAID databases. Therefore, the vaccines must include multi-variant protection, be immunogenic, and form immune memory. ^1^^,^^7^

Moreover, the development of vaccines involves the use of SARS-CoV-2 glycoprotein, which plays an important role in the infection process inside the host. ^7^ Under normal conditions, spike glycoprotein will bind to the angiotensin-converting enzyme 2 (ACE-2) receptor as a viral attachment mechanism. This mechanism has inspired many studies that have developed vaccines focusing on the neutralization of antibodies to viral glycoproteins. ^8^ In addition, SARS-CoV-2 is able to mutate to form a new variant that is capable of changing the residues which can create the spike glycoprotein. This leads to the possibility that the spike glycoprotein could avoid being neutralized by antibodies produced by SARS-CoV-2 vaccine. ^9^ However, a previous study suggested that the development of the SARS-CoV-2 vaccine must target the conserved regions. Conserved regions can be found in every variant of SARS-CoV-2, and are the main target of vaccine design because they are able to trigger an increase in the antibody neutralization’s coverage. ^10^

Additionally, conserved region-based vaccine development has been carried out on ZIKA, DENV and influenza viruses through an immunoinformatic approach, but is rarely found in SARS-CoV-2 vaccine development. ^11^ This approach was used in this study to determine the conserved region in the spike glycoprotein of Indonesian SARS-CoV-2 isolates, which can be used as vaccine seed candidates. BepiPred methods were employed in determining the B cell epitope to investigate the probability of the antigen region inducing the B cell recognition. ^12^ The candidate peptides that change the epitope of B cells would be expected to have antigenicity, similarity, and toxicity. Then, molecular docking and dynamic simulations would be carried out to determine the stability level of molecular complexes formed by peptides with B cell receptors (BCR). ^13^^,^^14^ Furthermore, this study aimed to investigate Indonesian SARS-CoV-2 isolates based on their B cell epitopes by targeting the conserved regions in spike glycoprotein to trigger the increased multi-variant virus neutralization and memory response to develop seed candidates.

## Methods

### Conserved region retrieval and 3D structure comparison

The SARS-CoV-2 spike glycoprotein gene sequences originating from Indonesian isolates and their references were obtained from NCBI and GISAID ^44^ databases with keyword ‘SARS-COV-2 Spike Glycoprotein’ and with filter ‘no partial sequences’.

Then, protein alignment was performed using MEGA X (version 10.2.6, build 10210527, default settings GAP, (Open/Extend: −2,90 & 0.00), Min Dag Length: 24, dan Max literation 16) to identify the position of the conserved region. ^1^^,^^15^ The SWISS-MODEL (default settings) was applied in this study for the construction of the 3D spike glycoprotein SARS-CoV-2 structure. It utilized the construction method of homology modelling by considering sequences only where >20% of residues were homologous, and model validation through the Ramachandran plot which only considered sequences considered if >90% of residues were in the favored region. ^16^^,^^17^ Additionally, comparisons of the simulation, staining selection, and structure of the SARS-CoV-2 query spike glycoprotein models with the reference sequences were completed using PyMol software version 2.5.0, ^18^ differentiating domain receptor, ligand and secondary protein structures by color.

### B cell linear epitope prediction

The recognition of antigens is performed directly by B cells through the BCR. This consists of immunoglobulin and Cluster of Differentiation (CD) 79, which majorly contribute to signal transduction and activation of B cells in the immune response. ^19^^,^^20^ The interaction between the variable region in the antigen-binding fragment (Fab) of the BCR and the epitope allow it to trigger the B cell recognition response, which can be predicted by epitope mapping analysis. ^21^^,^^22^ IEDB Server Analysis Tools (default settings) were employed in this study to predict B cell epitopes based on the direct recognition mechanism through the BepiPred version 2.0 method. ^8^^,^^12^^,^^23^

### CD4+ T cell epitope prediction

Indirect B cell activation occurs via the peptide recognition pathway by CD4+ T cells through the major histocompatibility complex (MHC)-II. Investigating binding of a peptide with human leukocyte antigens (HLA) can predict their ability to trigger the formation of memory cells. ^24^^,^^25^ MHC-II epitopes were predicted through IEDB’s binding tool (default settings, locus allel MHC type II: Human, HLA-DR, with epitope length 15-mer, sorted peptide by: adjusted rank, output format: XHTML table), by binding with three HLAs (reference set) consisting of HLA-DRB1*01:01, HLA-DRB4*01:01, and HLA-DRB3*02:02. ^26^ Meanwhile, the peptide binding affinity for MHC-II encoded with specific HLA alleles was predicted based on the IC _50_ value, which was determined through previous research by Zahroh *et al*. in 2011 ^45^; high affinity was represented by IC _50_ <50 nM, medium affinity by IC _50_ <500 nM, and low affinity by IC _50_ <5000 nM. ^11^

### Vaccine profiling and peptide modeling

Peptides with positive predictions (indicated by the IC _50_ value of the vaccine candidate peptide of <50 nm and the percentage rank value of <0.5) for B and T CD4+ cell epitopes were tested through the VaxiJen v2.0 server (Setting as = “target organism”: virus, threshold: default Output format: sequence Output) for the selection of antigenic properties. ^27^ Then, the level of allergenicity from the vaccine candidate peptides was predicted via the AlerCatPro 1.7 server (default settings). ^28^ The toxicity profile of the vaccine candidate peptides in this study was predicted using the ToxinPred server (threshold SVM: −0,1 with positive prediction (peptide but not toxin) indicated as peptide score below -0,1). ^29^ Lastly, the 3D peptide structure of the vaccine candidate was constructed via the PEP-FOLD 3.5 server (default settings) with *de novo* modeling methods, and a sample of the protein modeling result was saved in databank format. ^30^

### Molecular docking and dynamics simulation

The Cluspro 2.0 server (default settings) was applied in this study for molecular docking as the analysis aimed to determine the lowest amount of energy formed when the vaccine candidate peptide bound with BCR and MHC-II to create the molecular complex. The docking results were formed through superimposition. ^13^ Furthermore, the CABS-flex 2.0 server (default settings: protein rigidity: 1.0; protein restraints: ss2 3 3.8 8.0; global C-alpha restraints weight: 1.0; global side-chain restraints weight: 1.0; number of cycles: 50; cycles between trajectory: 50; temperature range: 1.40-1.40; RNG seed: 2465) was utilized in this study to validate the docking results through molecular dynamics simulation; when a molecular complex occurs, the dynamic molecular simulation can describe the fluctuations in the interaction of the amino acid residues that make up the peptide with the target protein domain (receptor). ^14^

## Results

### 3D structure modeling of Indonesian SARS-CoV-2 glycoprotein

The SARS-CoV-2 spike glycoprotein sequence of Indonesian isolates that were successfully obtained from the NCBI database had the following IDs: MZ026853.1, MZ026854.1, and MZ026855.1. Additional samples were also generated from the GISAID database, which can be found in the underlying data section. ^31^ These gene samples were then translated via MEGA X (version 10.2.6) software to identify the amino acid residues that formed the SARS-CoV-2 spike glycoprotein. The conserved region was obtained through protein alignment with reference sequences MN908947.3/Wuhan/China, MW980115.1/United Kingdom, MW981442.1/South Africa, MT434757.2/India, MT510724.1/United States, and MT807936.1/Brazil. The total number of spike glycoprotein gene sequences used in this study were 26 samples consisting of 20 queries and six reference sequences.

**Figure 1.**
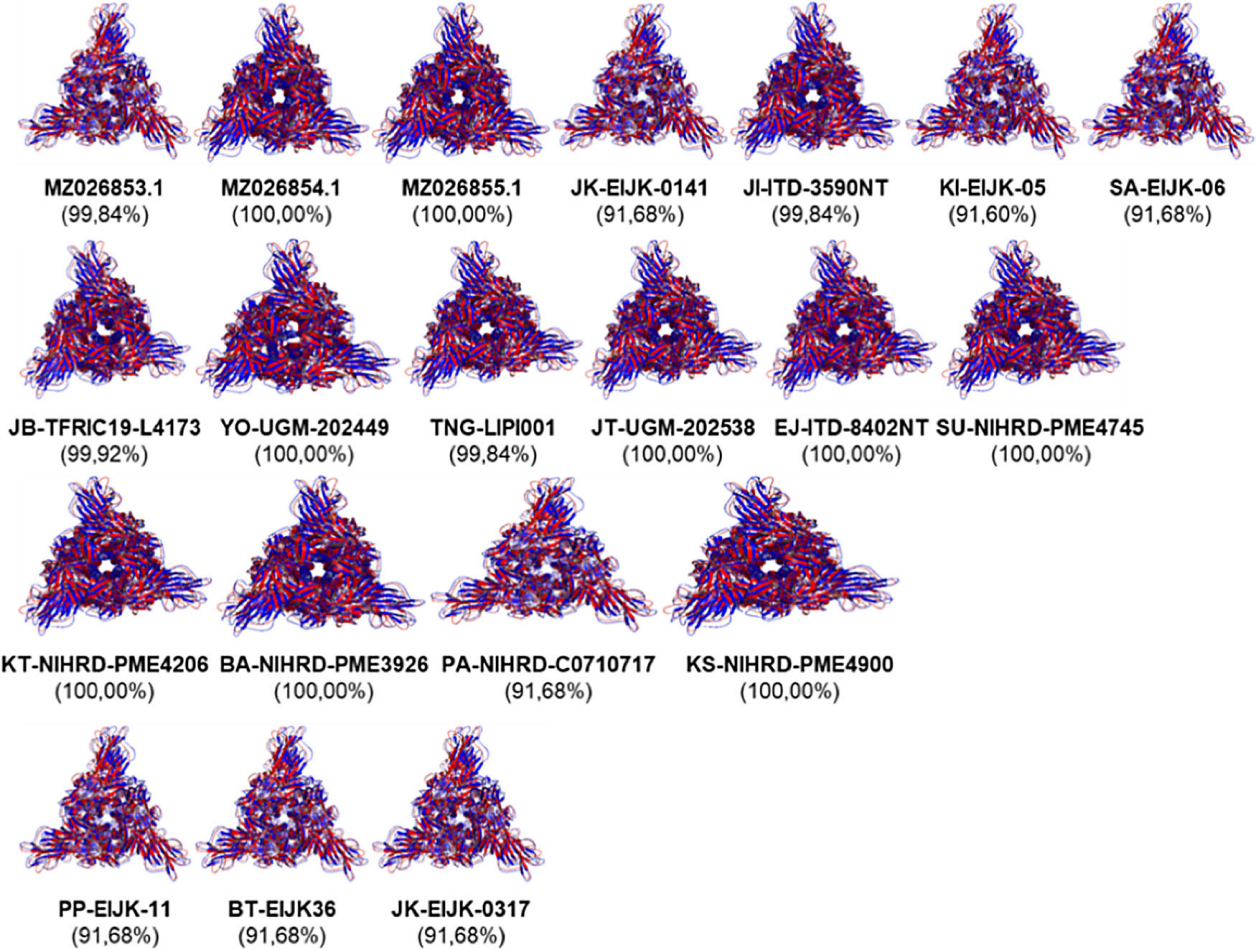
3D structure comparison of spike glycoprotein SARS-CoV-2 from Indonesian isolates (blue) with references (red). The structures are displayed in cartoons and colored based on the isolates via PyMol software.

The conserved region positions were identified as 18 regions in the SARS-CoV-2 spike glycoprotein consisting of A(1-17), B(23-68), C(81-115), D(117-143), E(145-184), F(186-214), G(216-240), H(244-476), I(485-500), J(502-569), K(571-613), L(615-671), M(683-700), N(702-715), O(717-981), P(988-1117), Q(1119-1139), and R(1141-1273). The 3D structures of spike glycoprotein isolates were obtained from SWISS-MODEL to determine the similarity to the structural templates and quality. The protein modeling results were displayed through the PyMol software with a cartoon display and underwent staining selection. When the results of the modeling of the spike glycoprotein SARS-CoV-2 query were completed, a comparison of the structure with the template was carried out through 3D alignment analysis. The resulting spike glycoprotein SARS-CoV-2 models of Indonesian isolates in this study had similarity values of 90-100% with the references in the database through the 3D alignment process ( Figure 1). ^32^

### Mapping of conserved B cell and T CD4+ epitope

**Figure 2.**
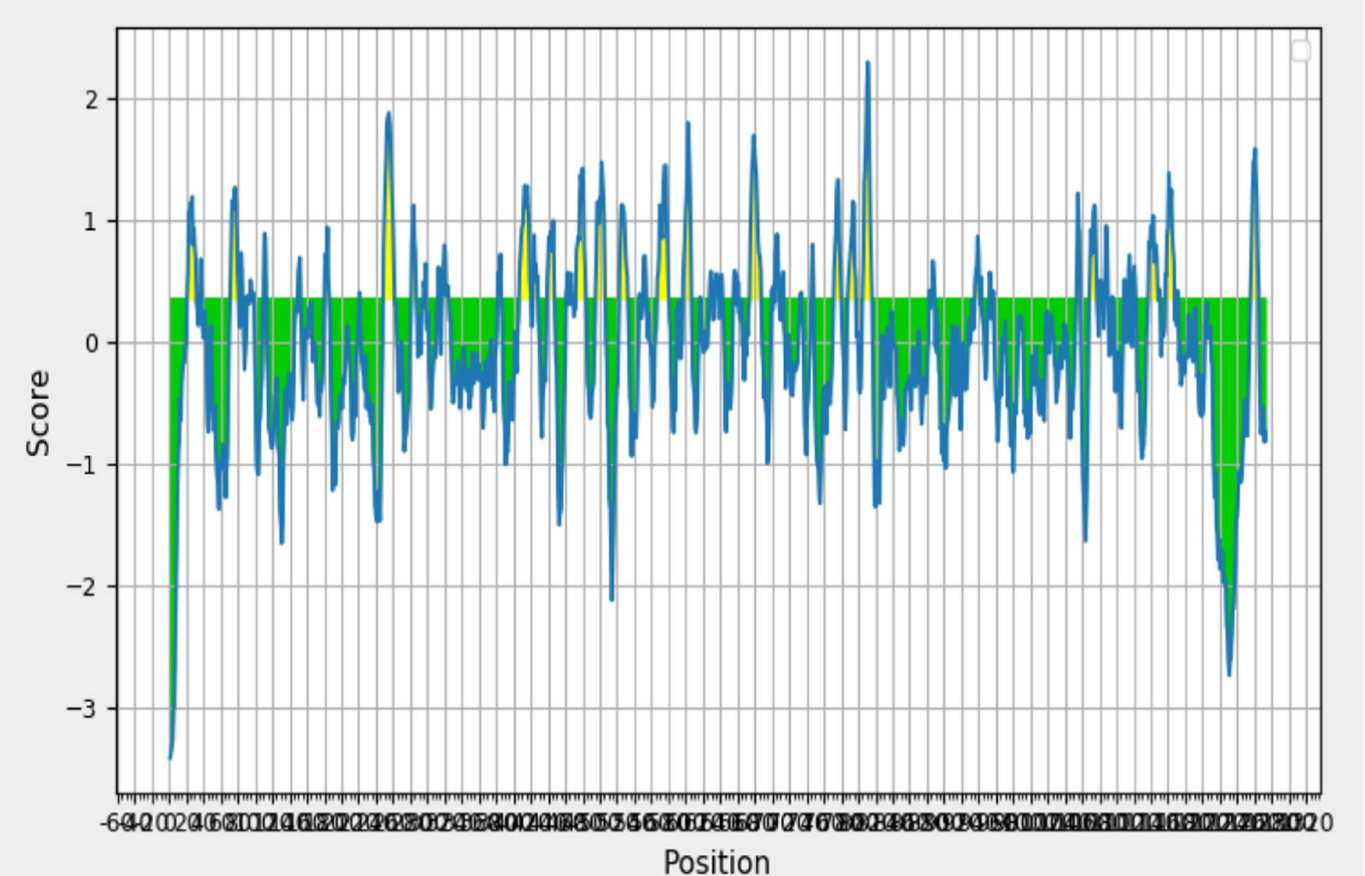
B-cell epitope prediction results on SARS-CoV-2 glycoprotein spike using BepiPred method. Regions with positive predictions are highlighted in yellow and green for negative predictions.

Indonesian isolate MZ026853.1 was chosen for SARS-COV-2 seed vaccine candidate as it is the dominant variant found in Indonesia. ^33^ Prediction of B cell epitope recognition was carried out on the template of Indonesian isolate MZ026853.1 using IEDB Analysis Tools with the BepiPred method. The peptides that showed a positive epitope prediction were selected based on the epitope’s carried conserved region. The epitope/peptide of the B cell-based vaccine candidate refers to a score above the threshold of 0.35 (calculated based on the server’s prediction results, which refers to the Hidden Markov Model and Trend Scale methods) while the T-cell-based vaccine candidate epitope/peptide refers to the IC _50_ score >50 nM. The results showed that the position of the B cell epitope was identified on the SARS-CoV-2 spike glycoprotein of the Indonesian isolate and had a length of 10 to 11-mer ( Figure 2). ^34^ Furthermore, the results also revealed that all identified epitopes had a value above the threshold of 0.35, and based on the identification results, it exhibited all peptides that make up the epitope. In addition, inside the obtained B cells epitope, there were eight peptides with conserved regions from the total of 15 epitopes in this study ( Table 1).

**Table 1.** Results of B cell epitope prediction of SARS-CoV-2 spike glycoprotein.

Peptide	Position	Sequence	Length (Mer)	Conserved region
B1	21-31	RTQLPPAYTNS	11	-
B2	71-81	SGTNGTKRFDN	11	-
B3	249-261	LTPGDSSSGWTAG	13	H
B4	407-420	VRQIAPGQTGKIAD	14	H
B5	473-483	YQAGSTPCNGV	11	-
B6	495-506	YGFQPTNGVGYQ	12	-
B7	523-532	TVCGPKKSTN	10	J
B8	567-580	RDIADTTDAVRDPQ	14	-
B9	597-606	VITPGTNTSN	10	K
B10	675-687	QTQTNSPRRARSV	13	-
B11	788-797	IYKTPPIKDF	10	O
B12	805-816	ILPDPSKPSKRS	12	O
B13	1137-1148	VYDPLQPELDSF	12	-
B14	1157-1167	KNHTSPDVDLG	11	R
B15	1256-1265	FDEDDSEPVL	10	R

The prediction of CD4+ T cell epitope introduction was also carried out on the template of Indonesian isolate MZ026853.1 using IEDB Analysis Tools with the default method. In addition, the binding affinity of the vaccine candidate peptide with MHC-II encoded by specific HLA alleles was assessed based on a reference set consisting of HLA-DRB1*01:01, HLA-DRB4*01:01, and HLA-DRB3*02:02. The results showed there were 15 peptides categorized as CD4+ T cell epitopes because they had a high binding affinity for MHC-II encoded by specific HLA alleles. Moreover, there were 11 peptides that produced epitopes identified as having conserved regions and these were utilized for further analysis ( Table 2).

**Table 2.** Prediction results of CD4+ T cell epitopes of SARS-CoV-2 spike glycoprotein.

HLA allele	23Position	Peptide	Sequence	IC50 score	Conserved region
DRB1*01:01	513-527	L1	LSFELLHAPATVCGP	2.80	J
	512-526	L2	VLSFELLHAPATVCG	2.70	J
	514-528	L3	SFELLHAPATVCGPK	3.30	J
	510-524	L4	VVVLSFELLHAPATV	3.30	J
	2-16	L5	FVFLVLLPLVSSQCV	43.10	A
DRB3*02:02	116-130	L6	SLLIVNNATNVVIKV	13.81	-
	114-128	L7	TQSLLIVNNATNVVI	16.27	-
	117-131	L8	LLIVNNATNVVIKVC	17.40	D
	1091-1105	L9	REGVFVSNGTHWFVT	25.75	P
	1093-1107	L10	GVFVSNGTHWFVTQR	30.09	P
DRB4*01:01	894-908	L11	LQIPFAMQMAYRFNG	22.20	O
	893-907	L12	ALQIPFAMQMAYRFN	24.10	O
	892-906	L13	AALQIPFAMQMAYRF	26.20	O
	231-245	L14	IGINITRFQTLLALH	30.30	-
	5-19	L15	LVLLPLVSSQCVNLT	31.90	-

### Vaccine properties prediction

There were 19 peptides which have B and T CD4+ cells with conserved regions. These were then tested for antigenicity considering the threshold value of 0.4 (determined by IEDB’s default settings), which led to 13 peptides categorized as antigens because they had values higher than this. Furthermore, peptides with antigenic properties were tested for allergenicity by taking into account the threshold value of 20% (determined by IEDB’s default settings), which resulted in all 13 obtained peptides being considered non-allergenic. Peptides categorized as non-allergenic then underwent toxicity prediction using the reference sequences with a threshold value of −0.8 (determined by IEDB’s default settings), which incurred 10 non-toxic peptides ( Table 3). Thus, based on the analysis of vaccine property prediction, it was concluded that peptides B9, B12, L1, L2, L4, L5, L9, L11, L12, and L13 could act as antigens and were not allergenic, as well as having non-toxic properties. These 10 peptides were then used for further analysis.

**Table 3.** Prediction results of SARS-CoV-2 vaccine candidate peptide properties.

Peptide	Antigenicity (>0.4)		Allergenicity (<20%)		Toxicity (<−0.8)	
	Score	Properties	Score	Properties	Score	Properties
B3	0.49	Antigen	0%	Non-allergen	−0.78	Toxin
B4	1.26	Antigen	0%	Non-allergen	−0.78	Toxin
B7	−0.02	Non-antigen	-	-	-	-
B9	0.42	Antigen	0%	Non-allergen	−1.32	Non-toxin
B11	−0.16	Non-antigen	-	-	-	-
B12	0.53	Antigen	0%	Non-allergen	−0.88	Non-toxin
B14	1.40	Antigen	0%	Non-allergen	−0.41	Toxin
B15	0.31	Non-antigen	-	-	-	-
L1	0.50	Antigen	0%	Non-allergen	−1.10	Non-toxin
L2	0.47	Antigen	0%	Non-allergen	−1.31	Non-toxin
L3	0.20	Non-antigen	-	-	-	-
L4	0.83	Antigen	0%	Non-allergen	−1.49	Non-toxin
L5	0.71	Antigen	0%	Non-allergen	−1.06	Non-toxin
L8	0.09	Non-antigen	-	-	-	-
L9	0.44	Antigen	0%	Non-allergen	1.02	Non-toxin
L10	0.32	Non-antigen	-	-	-	-
L11	0.72	Antigen	0%	Non-allergen	−1.19	Non-toxin
L12	1.01	Antigen	0%	Non-allergen	−1.16	Non-toxin
L13	0.91	Antigen	0%	Non-allergen	−1.18	Non-toxin

### Peptide-protein docking and dynamic simulation

**Figure 3.**
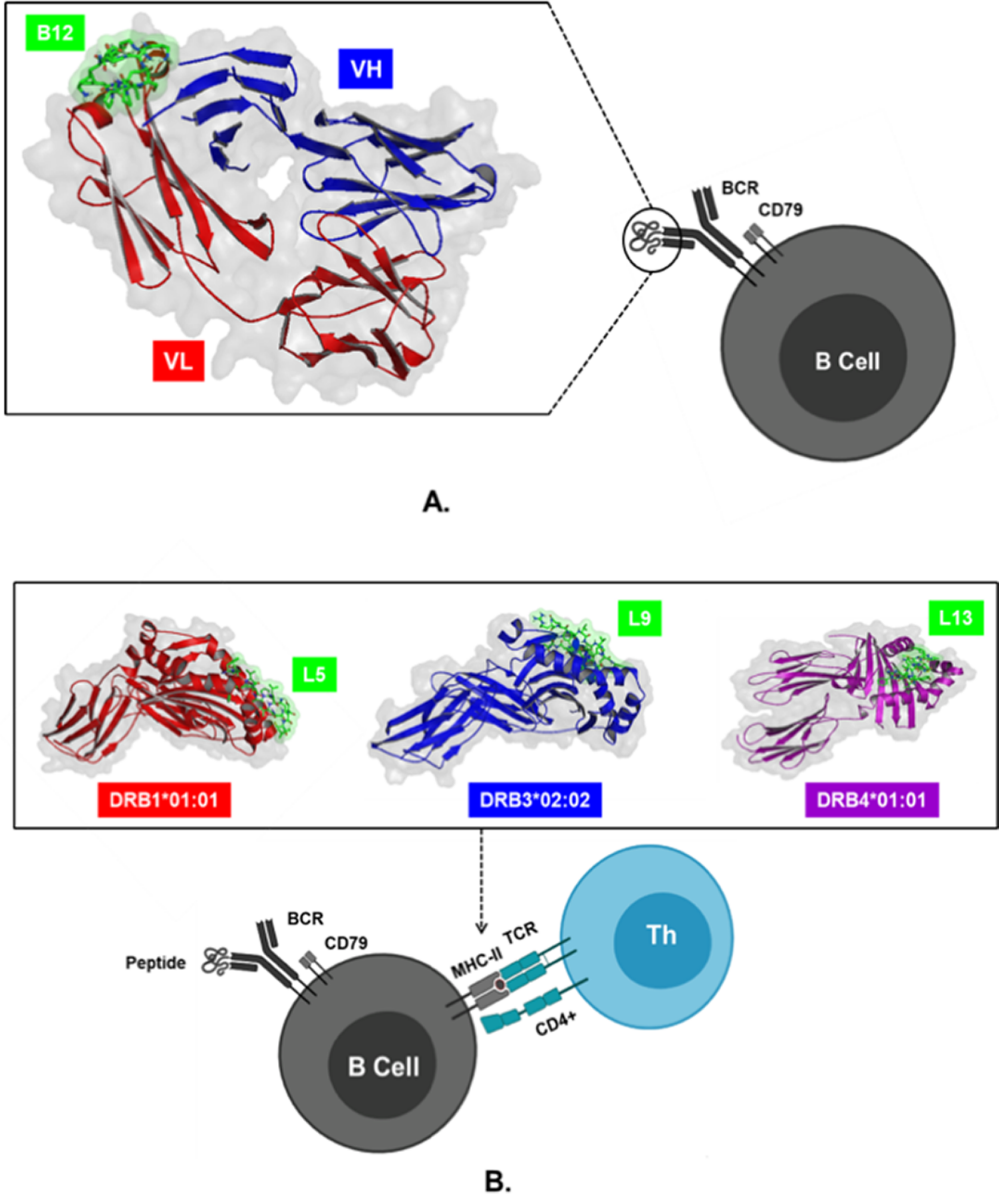
Visualization of peptide-protein docking results. (A) Interaction of B12 peptides with variable light (VL) and heavy (VH) chains on the B cell receptor (BCR) through direct B cell activation pathways. (B) Interaction of L5, L9, and L13 peptides bound to region A on MHC-II encoded by HLA-II to trigger activation B cells through an indirect mechanism with the help of CD4+ T cells.

The 3D structures of BCR (ID: 5IFH), HLA-DRB1*01:01 (ID: 1AQD), HLA-DRB3*02:02 (ID: 3C5J), and DRB4*01:01 (ID: 5JLZ) were obtained from RCSB Protein Data Bank. 3D peptide structures of B9, B12, L1, L2, L4, L5, L9, L11, L12, and L13were modelled using the PEP-FOLD 3.5 server. The structures of peptides and proteins were used for docking simulations on Cluspro 2.0 software to determine the lowest energy formed in the molecular complex. The docking models in this study were peptides B9 and B11 which interacted with BCR, peptides L1, L2, L4, L5 with MHC-II coded by HLA-DRB1*01:01, peptides L9 with HLA-DRB3*02:02, and peptides L11, L12, and L13 with HLA-DRB4*01:01. The simulation results showed that the molecular complex BCR_B12 with the lowest energy of -433.0 kcal/mol was more negative than B9 when binding to BCR. Meanwhile, peptide L5 had the lowest energy -905, 6 kcal/mol when interacting with MHC-II encoded by HLA-DRB1*01:01, which was lower than L1, L2, and L4. The most negative values occurred in the interaction between peptide L9 and HLA-DRB3*02:02, which had an energy value of -890.6 kcal/mol, and L13 peptide, which possessed lower energy (-789.3 kcal/mol) compared to L11 and L12 when binding to HLA-DRB4*01:01 ( Figure 3). ^35^

**Figure 4.**
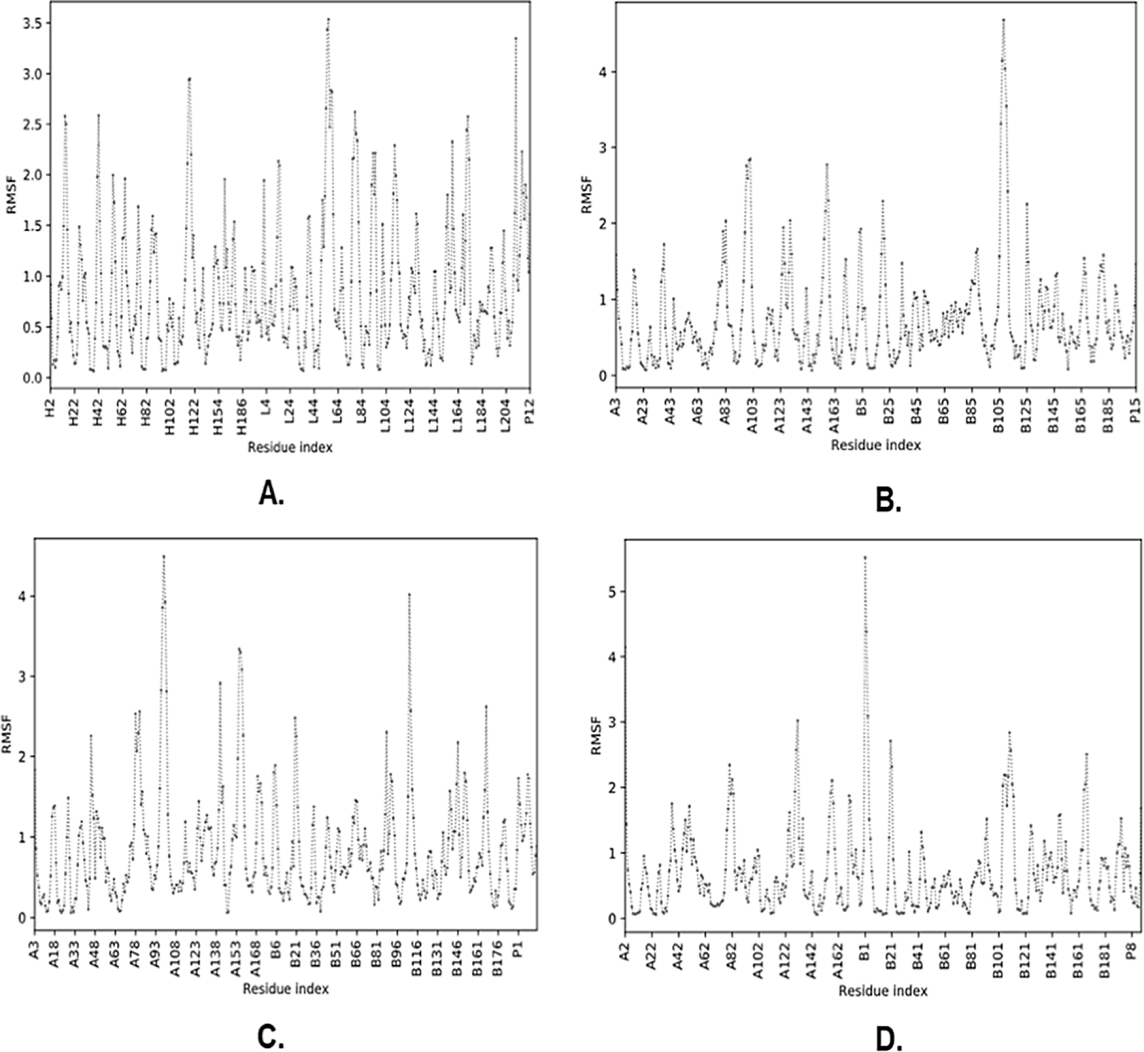
Root-mean-square fluctuation (RMSF) graph of peptide-protein complex as a result of molecular dynamic simulation. The residues that produce L and H chains are part of the BCR, while chain A is presented in MHC-II, and P is the vaccine candidate peptide. (A) BCR_B12, (B) HLA-DRB1*01:01_L5, (C) HLA-DRB3*02:02_L9, (D) HLA-DRB4*01:01_L13.

The molecular complexes of BCR_B12, HLA-DRB1*01:01_L5, HLA-DRB3*02: 02_L9, and HLA-DRB4*01:01_L13 were analyzed for residual fluctuations in their peptide constituent that interacted through root-mean-square fluctuation (RMSF) level and generated the protein region via the CABS-flex 2.0 server. The results of molecular dynamic simulations exhibited that the BCR_B12 molecular complex contained a fluctuating residue with positions A3, A4, A5, A6, A7, A8, A9, and A10 with RMSF values >0.85 Å to <2.22 Å. Concurrently, the molecular complex of HLA-DRB1*01:01_L5 showed fluctuant residue positions of A9, A10, A11, A12, A13, A14, and A15 with RMSF values >0.52 Å to <1.46 Å. Furthermore, the HLA-DRB3*02:02_L9 molecular complex showed fluctuant residue positions of A4, A5, A6, A7, and A8 with RMSF values ranging from >0.95 Å to <1.77 Å. Additionally, the molecular complex of HLA-DRB4* 01: 01_L13 showed fluctuant residues at positions A11, A12, A13, A14, and A15 with RMSF values ranging from >0.46 Å to <1.68 Å ( Figure 4). ^37^

## Discussion

Homology modeling is a method of determining the 3D structure of a query protein based on the availability of templates in the database. Its accuracy is around 90%, and the model is categorized as similar or homologous to the target protein if it has a modeling similarity value of >20%. ^16^^,^^17^ The similarity levels are high if the superimposed query protein on the template is identified during 3D alignment. ^18^^,^^38^ In this study, the spike glycoprotein structure of 20 Indonesian isolates of SARS-CoV-2 had high similarities to the isolate references such as those from China/Wuhan, the United Kingdom, South Africa, India, the United States, and Brazil with an average similarity level of 90% to 100% (See Figure 1). In addition, the similarities were also demonstrated between the secondary protein structure of the SARS-CoV-2 spike glycoprotein of the Indonesian isolates and the superimposed reference isolates.

The structure of the SARS-CoV-2 spike glycoprotein consists of S1 and S2 regions. S1 covers residue positions 13-685 and S2 covers positions 686-1273. ^39^ In the S1 region, there is a N-terminal domain as well as a receptor-binding domain (RBD) covering the sequence positions 319-541, including an integrin-binding motif (403-405), and receptor-binding motif (437-508). S2 consists of fusion peptides 1 and 2 (816-1202). ^40^ The conserved region is specific to the virus, is maintained in each generation of variants and has the potential to be a target for antibodies to neutralize of in vaccine design, since it is predicted to be able to protect against infections with a wide variety of variants. ^8^^,^^13^^,^^41^ Furthermore, the conserved region obtained from the identification results in this study was detected very high activity in almost all parts of the SARS-CoV-2 spike glycoprotein, especially in S1, RBD, and S2.

The recognition of B cell epitopes in the conserved region allows activation of B cells through direct and indirect pathways ^19^^,^^20^ The epitope position on the SARS-CoV-2 spike glycoprotein recognized by B cells based on the study results can be influenced by the direct activation of conserved regions H, K, O, & R. This allows the production of Immunoglobulin M (IgM)-type immunoglobulins for opsonization response and complement recruitment. ^42^^,^^43^ In addition, conserved regions A, J, D, P, O may trigger B cell activation through indirect pathways, through the contribution of CD4+ T cells to produce isotype switching for the production of IgG antibodies and memory cells. ^44, 45^ Correspondingly, the peptides with specific regions were B9 (region K), B12 (region O), L1 (region J), L2 (region J), L4 (region J), L5 (region A), L9 (region P), L11 (region O), L12 (region O), and L13 (region O) including antigenic peptides, non-allergens, and non-toxins.

The direct B cell activation pathway is triggered by peptide binding to the Fab region on the BCR, which allows signal transduction through CD79, and then triggers transcription factors involved in the activation, maturation, and proliferation of plasma cells, resulting in the production of IgM antibodies. ^19,46^ On the other hand, the indirect pathway of activation occurs due to the representation of MHC-II by B cells, and its recognition by TCR on CD4+ T cells. ^20,47^ Peptides with high binding affinity to MHC-II and encoded by specific HLA alleles are predicted to act as T cell epitopes. ^11^ The binding of the vaccine candidate peptide to the B cell receptor is identified by the lowest energy produced by the molecular docking complex. The lowest energy with a more negative value, is estimated to indicate the most stable and active biological response to protein initiation. ^13^ In this study, the activation response of B cells is predicted to be directly initiated by the molecular complex BCR_B12, and indirectly through HLA-DRB1*01:01_L5, HLA-DRB3*02:02_L9, and HLA-DRB4*01:01_L13 because they have the lowest negative energy values.

Finally, the binding stability of molecular complexes can be analyzed by molecular dynamics simulations by examining the level of fluctuation based on the RMSF value of each complex formed. ^14^ RMSF exhibits the movement of interacting atoms on the residues of peptides and proteins with a certain distance, while complex stability is achieved when the resulting distance has a value of ≤4Å. ^48,49^

## Conclusions

The spike glycoprotein sequences of Indonesian SARS-CoV-2 isolates have a conserved region and are very similar to the reference isolates from China, the United Kingdom, South Africa, India, the United States, and Brazil. It is suggested that the conserved region of Indonesian SARS-CoV-2 isolates can be identified as an epitope of B and T CD4+ cells that produce the vaccine candidate peptides with antigenic, non-allergen, and non-toxic properties. This study recommends that peptides B12, L5, L9, and L13 can be used in the follow-up tests because they may trigger B cell activation responses via direct and indirect pathways. Lastly, the vaccine candidate is predicted to be able to initiate the production of specific antibody isotypes, which play a role in increasing the neutralization of multi-variant viruses and the formation of memory cells to prevent SARS-CoV-2 infection.

## Data availability

### Underlying data

The accession numbers for GISAID and NCBI can be found in the acknowledgments table:

Figshare: GISAID acknowledgment table: https://doi.org/10.6084/m9.figshare.15048513.v1. ^31^

Access to GISAID data requires registration and agreement to GISAID’s terms.

Data are available under the terms of the Creative Commons Attribution 4.0 International license (CC-BY 4.0).
