# Supplementary material for: Molecular docking and dynamic simulation of conserved B cell epitope of SARS-CoV-2 glycoprotein Indonesian isolates: an immunoinformatic approach
Source: F1000Res. 2021 Aug 16;10:Chem Inf Sci-813. [Version 1] doi: 10.12688/f1000research.54258.1 (PMC8596179; doi:10.12688/f1000research.54258.1)
Supplement: Peer review history

# Peer review: Reviewer response for version 1

**DOI:** 10.5256/f1000research.57725.r98417

**Published:** 2021-11-16

**Authors:** Hesham Mohammed Al-Sharani

**Affiliations:** 1 Faculty of Dentistry, Ibb University, Ibb, Yemen

The study entitled "Molecular docking and dynamic simulation of conserved B cell epitope of SARS-CoV-2 glycoprotein Indonesian isolates: an immunoinformatic approach" was an original article that is well organized and valuable. It checked the possibility of investigating SARS-CoV-2 in Indonesian isolates based on B cell epitopes by marking the conserved regions in the spike glycoprotein to trigger the elevated multi-variant virus neutralization and memory response for the development of vaccine seed candidates.

 Methods included a comparison between SARS-CoV-2 spike glycoprotein gene sequences originating from Indonesia with Wuhan (China), the United Kingdom, South Africa, India, the United States, and Brazil isolates collected from the NCBI and GISAID databases. B cell receptor (BCR) was used to recognize antigens on the B cells. Indirect B cell activation by Cluster of Differentiation (CD)4+ T cells and major histocompatibility complex (MHC)-II was predicted through the binding with human leukocyte antigen (HLA) based on IC50 value. Vaccine allergenicity and toxicity were also examined. 

 Results showed similarities between Indonesian conserved regions and the compared mentioned countries, thus the predicted expectations that the conserved regions could be identified as the epitope of B and T CD4+ cells that produced the peptides for vaccine candidates with antigenic, non-allergen, and non-toxic properties.

 I feel satisfied with the article's frame, methodology and results.

 Just a few English corrections are to be rechecked.

Is the work clearly and accurately presented and does it cite the current literature?

Yes

If applicable, is the statistical analysis and its interpretation appropriate?

Yes

Are all the source data underlying the results available to ensure full reproducibility?

Yes

Is the study design appropriate and is the work technically sound?

Yes

Are the conclusions drawn adequately supported by the results?

Yes

Are sufficient details of methods and analysis provided to allow replication by others?

Yes

Reviewer Expertise:

Clinical and lab studies in medicine and dentistry. Systematic reviews and meta-analyses as well.

I confirm that I have read this submission and believe that I have an appropriate level of expertise to confirm that it is of an acceptable scientific standard.

# Peer review: Reviewer response for version 1

**DOI:** 10.5256/f1000research.57725.r98953

**Published:** 2021-11-16

**Authors:** Dur Lashari, Yasmeen Lashari

**Affiliations:** 1 Department of Oral Biology, Bolan University of Medical and Health Sciences, Quetta, Pakistan; 2 Bolan University of Medical and Health Sciences , Quetta, Pakistan

The current study, “Molecular docking and dynamic simulation of conserved B cell epitope of SARS-CoV-2 glycoprotein Indonesian isolates: an immunoinformatic approach,” which employs an Immunoformatics approach, appears to be a high-quality piece of work with promising results. The use of bioinformatics tools reduces the cost and time required to predict potential epitopes. Furthermore, the current study is appealing as a first step in developing a vaccine because it focuses on the local Indonesian sequences of SARS-CoV-2.

 I recommend this study strongly for indexing due to the following reasons:

- The current study is well-presented, and the most recent literature is cited.
- The study design was found sound and used immunoinformatic approaches for B-cell, T-cell prediction, 3D structure development, and molecular docking are technically sound approaches.
- The methodology and the discussion section were both clear, explanatory, and technically sound.
- The study's abstract and conclusion were found to be brief and concise.

Is the work clearly and accurately presented and does it cite the current literature?

Yes

If applicable, is the statistical analysis and its interpretation appropriate?

I cannot comment. A qualified statistician is required.

Are all the source data underlying the results available to ensure full reproducibility?

Yes

Is the study design appropriate and is the work technically sound?

Yes

Are the conclusions drawn adequately supported by the results?

Partly

Are sufficient details of methods and analysis provided to allow replication by others?

Yes

Reviewer Expertise:

immunology and microbiology

We confirm that we have read this submission and believe that we have an appropriate level of expertise to confirm that it is of an acceptable scientific standard.

# Peer review: Reviewer response for version 1

**DOI:** 10.5256/f1000research.57725.r91996

**Published:** 2021-09-09

**Authors:** Mohammad Saifur Rohman

**Affiliations:** 1 Cardiovascular Research Group, Faculty of Medicine, Brawijaya University, Malang, Indonesia

This manuscript is promising for initial step of vaccine development. Especially for Indonesian population. Since isolated sequence taken from Indonesian covid-19 patients.

- However, as preliminary result the author supposed to propose future direction of the next research for vaccine production.
- Molecular docking and dynamic simulation results should be confirm again using in vitro or in vivo models. It could be proven or not according to this result. It is far for realistic the candidate of molecule and the rapid test really occur in vivo.

Is the work clearly and accurately presented and does it cite the current literature?

Yes

If applicable, is the statistical analysis and its interpretation appropriate?

Yes

Are all the source data underlying the results available to ensure full reproducibility?

Yes

Is the study design appropriate and is the work technically sound?

Yes

Are the conclusions drawn adequately supported by the results?

Yes

Are sufficient details of methods and analysis provided to allow replication by others?

Yes

Reviewer Expertise:

Cardiovascular Medicine, Molecular Cardiology, Interventional Cardiology, Pharmacogenetics

I confirm that I have read this submission and believe that I have an appropriate level of expertise to confirm that it is of an acceptable scientific standard.
